# Streamlined Operational Approaches and Use of e-Technologies in Clinical Trials: Beat Acute Myeloid Leukemia Master Trial

**DOI:** 10.1007/s43441-021-00277-w

**Published:** 2021-05-16

**Authors:** Len Rosenberg, Hugh Levaux, Ross L. Levine, Amit Shah, James Denmark, Nyla Hereema, Melanie Owen, Spencer Kalk, Nicholas Kenny, Gene Vinson, Jo-Anne Vergilio, Alice Mims, Uma Borate, William Blum, Eytan Stein, Theophilus J. Gana, Mona Stefanos, Ashley Yocum, Sonja Marcus, Abigail Shoben, Brian Druker, John Byrd, Amy Burd

**Affiliations:** 1 The Leukemia & Lymphoma Society, Leukemia & Lymphoma Society (National Office), 3 International Drive, Suite 200, Rye Brook, NY 10573 USA; 2 Protocol First, Salt Lake City, USA; 3 Memorial Sloan Kettering Cancer Center, New York, USA; 4 myClin, Yardley, USA; 5 The Ohio State University James Cancer Center, Columbus, USA; 6 Syneos Health, Morrisville, USA; 7 Foundation Medicine, Cambridge, USA; 8 The Ohio State University James Cancer Center, Columbus, USA; 9 Emory University Winship Cancer Institute, Atlanta, Georgia

**Keywords:** Clinical trials, E-technologies, EDC, Remote monitoring, EHR-to-EDC, Artificial intelligence

## Abstract

Advances in genomic technologies and an increased understanding of the molecular pathogenesis of cancer have resulted in development of new effective, mutation-targeted therapies. In turn, these informed the development of Master Trial designs to test these therapies. The Beat Acute Myeloid Leukemia (BAML) Master Trial (Sponsor: The Leukemia & Lymphoma Society) tests several targeted therapies in patients aged ≥ 60 years with AML based on genomic profiling obtained within 7 days of study enrollment. We hypothesized that integrating operational strategies with new electronic technologies (e-technologies) might streamline the conduct and management of this Master Trial. BAML’s 5 core operational strategies revolve around the guiding principle of “patients first.” The e-technology platforms employed in BAML include: Clinical Oversight Platform: a central collaborative tool; e-Protocol/e-Source Upload/Electronic Data Capture Platform: digitizes the protocol, allows remote data monitoring, and collects/exports data in Study Data Tabulation Model format; and Data Review Platform: ingests data from different sources for clinical response and safety data reviews. The operational approaches, e-technologies and sponsor/contract research organization’s (CRO) expertise together allow: the complexity and size of the BAML Master Trial to be better managed; near real-time study data oversight; better collaboration, communication and training; improved data collection, enhanced transmission and accessibility; data integration, review and generation of reports; while maintaining data privacy, and compliance. Initial e-technology challenges were overcome through training, learning, discipline and adjustment. In conclusion, to successfully manage Master Trials, significant time should be spent re-evaluating, improving and developing new operational approaches.

**Clinical Trial Registration:** Clinical Trials.gov Identifier: NCT03013998. https://clinicaltrials.gov/ct2/show/NCT03013998.

## Introduction

Advances in genomic technologies and in our understanding of cancer genetics has led to an explosion of targeted therapies and there is growing evidence that patients benefit from this paradigm shift, often termed as personalized medicine [1–3]. The challenge for clinical researchers and for drug development in cancer therapeutics is how to apply precision-based enrollment to evaluate new targeted therapies in an efficient manner for the subsets of patients with rare mutations, each of which often may represent 10% or less of the overall patient population. The resultant evolution of clinical trial designs to include master clinical trials or protocols [4] are seen as a critical approach to ethically and efficiently test molecularly targeted therapies in specific patient populations [5]. Multiple examples of such trials have demonstrated, on a national scale, that next-generation sequencing (NGS) can be used to make treatment decisions [6–9].

A master protocol (MP) is a protocol designed with multiple sub-studies, which may have different objectives and involves coordinated efforts to evaluate one or more investigational drugs in one or more disease subtypes within the overall trial structure [5]. There are three types of specific MP study designs and they include basket, umbrella, or platform trials [4, 5, 10]. The BRAF-V600 study [11], LUNG-MAP [7, 12] and I-SPY 2 [8] are examples of basket, umbrella, and platform trials, respectively. Master protocols have been more widely used in oncology because advances in identifying tumor mutations have advanced cancer research in precision targeting of treatments [4]. However, interest in using MPs in other non-oncology therapeutic areas has been growing, as in oncology. Reviews of MPs, their inherent benefits, efficiencies and challenges are provided elsewhere [13–17].

Master protocols in reality are 10 or more unique studies “disguised” as one study and require arduous information flow with safety and efficacy evaluations that happen early and often, operational nimbleness, access to data in real- or near real-time, and the ability to quickly evaluate patient and protocol progress, ultimately unifying trial governance and optimizing oversight. Hence, innovative ways for administration and oversight of Master Trials must be continuously sought.

Over the past decade, there has been a rapid growth in the use of digital and electronic technologies (e-technologies) in clinical trials. The e-clinical solutions market, including internet- and cloud-based solutions, is projected to grow at a compound annual growth rate of 12% such that it exceeds more than US $9.0 billion by 2024 [18]. This growth is having a significant ongoing impact on every aspect of the clinical trial process spanning trial design, protocol development, patient recruitment, study start-up, trial administration, and data collection and dissemination. The use of e-technologies in clinical trials started with the advent of electronic data capture but has now expanded to include electronic trial master file (e-TMF), electronic source documentation (e-source), and risk-based monitoring, all of which have been boosted by the recent introduction of cloud-based technologies and most recently by the introduction of artificial intelligence (AI)-based technologies [19–21]. Detailed reviews of the different types of digital and e-technologies currently being used in clinical trials can be found elsewhere in several recent reviews [19–24].

Due to the complexity, workload, size and skills required to manage Master Trials, we hypothesized that a combination of modified traditional operational strategies and new e-technologies may streamline and improve data collection, enhance transmission and accessibility, and optimize oversight. This paper reports the use of e-technologies in the design and implementation of MPs in cancer clinical trials, specifically the Beat Acute Myeloid Leukemia (BAML) Master Trial, the guiding principles, operational strategies, potential advantages, and the challenges we encountered with our resultant solutions.

## The Beat AML Master Clinical Trial

The Leukemia & Lymphoma Society (LLS), a patient-focused charitable (501[c] [3]) organization, is a world leader in the fight to research and cure blood cancers. LLS met with key opinion leaders in 2014 to discuss the lack of progress in developing new therapeutic approaches in AML and the urgent medical need for action as the standard of care had not changed in over 40 years. AML is a hematologic malignancy with mutational heterogeneity and different genetic subtypes, making it difficult to develop treatments that work for patients regardless of genotype. The lack of progress in developing new therapeutic options for AML is due, at least in part, to its complexity as it is now known to represent at least 10–12 diseases based on molecular characterization rather than one disease. Furthermore, the majority of new drugs are evaluated in patients with relapsed/refractory disease, where there is extensive genetic and epigenetic evolution, making clinical trials less likely to demonstrate efficacy. Thus, treatment earlier when the disease is less biologically complex, when patients have not experienced side effects from intensive therapy, and their immune system is less compromised, offers a chance for a better outcome. This coupled with the increasing evidence of efficacy for targeted therapies in AML, led LLS and the key opinion leaders to hypothesize: could we improve patient outcomes by matching patients to appropriate therapy based on genetics?

Therefore, LLS developed the BAML Master Trial, a collaborative trial between LLS, multiple academic medical research centers, biopharmaceutical companies, a contract research organization (CRO) and the FDA, with the objective of testing several novel targeted therapies for AML patients [25]. The BAML trial is designed as an umbrella study that requires screening upon entry. The overall primary endpoint of the trial is the feasibility of making a central treatment decision based on cytogenetics and NGS within 7 days. By using integrated analysis of metaphase cytogenetics done locally at the clinical site, NGS done centrally at Foundation Medicine, and FLT3-ITD mutation analysis performed at Invivoscribe to identify somatic alterations that contribute to AML pathogenesis and therapeutic response, the study stratifies patients based on their genomics. Given the rapid kinetic of AML disease course, treatment has historically been initiated immediately. However, a retrospective analysis suggests that a delay in treatment for up to 8 days does not influence overall survival in AML [26], providing support for the 7-day window for genetic characterization before initiating treatment in BAML.

**Figure 1:**
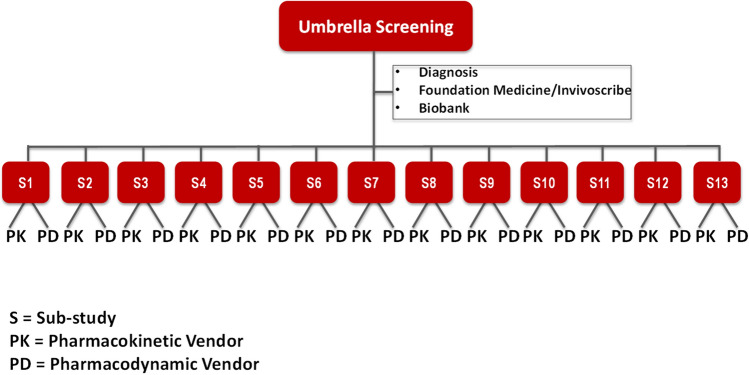
Schematic of Beat AML flow of information, and of patients into the Sub-Studies, and the flow of pharmacokinetic samples. Following screening (cytogenetics and next-generation sequencing) and central treatment decision, patients were enrolled into the 13 sub-studies where they were treated with one of the 9 investigational targeted therapies as monotherapy and/or combination therapy with either azacitidine or decitabine

Following patient screening using cytogenetics and NGS, once a central treatment decision is made, patients are enrolled into 1 of 13 sub-studies investigating 9 investigational drugs (Fig. 1). Each sub-study is designed as a traditional stand-alone study where efficacy, safety, pharmacokinetics and pharmacodynamics are all evaluated. Because each drug’s activity is driven by a different mechanism of action, each sub-study has different primary and secondary endpoints, different design and schedule of assessments, and each requires its own statistical analysis plan. Figure 1 illustrates the complexity of the trial by mapping the flow of patients, pharmacokinetic blood samples and information to support the 13 sub-studies.

As of February 28, 2021, the Beat AML study is open at 16 leading academic medical centers across the United States, has 13 sub-studies, involves 9 biopharmaceutical partners, and has enrolled over 1,065 patients in 4.33 years.

BAML Master Trial was designed to permit an iterative process wherein sub-studies (protocols) can either be added or removed throughout the life of the overall study as new drugs and new data become available. The individual sub-studies/protocols are designed to achieve a large clinical benefit and therefore, the trial utilizes a synthetic control arm as the comparator [25].

## Guiding Principles of the Beat AML Master Trial

The guiding principles for the BAML Trial established from the onset because of the collaborative nature of the trial include: (i) patients first; (ii) investigator development; (iii) communication and transparency; and (iv) dedication.

Patients First: This fundamental principle is focused on what is best for the patient and the therapeutic area of AML, and not about seeking individual academic credit for success. In addition, for presentations, abstracts, and manuscripts, authorship rules were established around extent of scientific input and not based on patient accrual.

Investigator Development: Because 100% of the collaborating study sites for the BAML Master Trial are academic medical centers, each sub-study protocol would have an assigned principal investigator that was a junior investigator and a co-principal investigator that was a senior investigator from the participating sites. The intent was to engage all investigators in trial design and execution, and to establish a method for mentoring and training the next generation of clinical investigators.

Communication and Transparency: Between partners this will be top priority to assure rapid implementation and completion of the project.

Dedication: Lastly, prioritization of individual scientific and institutional resources of the partners to this effort will occur to further assure rapid implementation and the attainment of key milestones of the project.

## Overall Strategy to Handle Operational Complexity

At inception, the 5 core operational strategies articulated to support the “patients first” principle were: (1) start small, learn and then expand; (2) centralize and standardize; (3) continuously evaluate and improve; (4) expertise and quality (in service delivery); and, (5) embrace new technologies.

### Start Small

To address and navigate the operational complexity presented by the BAML trial, the study initially started with 3 sites and three sub-studies (Ohio State University [OSU], Oregon Health & Science University and Memorial Sloan Kettering Cancer Center). With investigators at the initial 3 sites, the trial team worked through issues surrounding contracting, IRB approval, site initiation visits, validation of approach and workflow surrounding centralized NGS within the prescribed 7-day period, roll-out of new technology solutions and associated training, etc. Thereafter, we expanded the trial to more sites and sub-studies.

### Centralize & Standardize

Given the number of stakeholders and range of activities involved, from the beginning of the study we centralized several activities within BAML including, contracting and budgeting for both the sites and biopharmaceutical companies, Institutional Review Board (IRB) review, and scientific review (SR). BAML chose from the onset standard terms for contracting and budgeting to ensure near uniformity of terms across all institutions and biopharmaceutical companies allowing them to all work on a level-footing and without localized incentives that might distort the program as a whole.

IRB reviews by individual institutions can result in significant delay in trial review and startups, which can be compounded for studies with multiple amendments per year. While every academic center maintains some level of local review, a central IRB is used to provide a streamlined oversight of the trial and also consistency for the clinical sites, instead of a host of individual local committees. Additionally, most central IRB reviews are completed expeditiously (2 weeks median time for review).

Most cancer centers conduct SRs where the investigators vet the scientific integrity of the protocol and may require changes, as part of maintaining the National Cancer Institute (NCI) Cancer Center accreditation. This review if conducted at every site can be time-consuming and may require protocol amendments to accommodate requested changes. BAML has streamlined the process by utilizing a central SR that is conducted at the lead clinical site, OSU, and is accepted by the NCI, allowing for rapid reviews and decision making. Overall, the use of standard contracting, and central IRB and SRs have all combined to streamline operations.

It is important to stress here that BAML was able to employ centralized functionalities in part because of its focus on data privacy. The e-technologies presented below offer outstanding protection for patient and data privacy, thanks to their state-of-the-art architecture, strong data encryption in-transit and at-rest, as well as access rights that offer role-based access to specific data types.

### Continuously Evaluate and Improve

At inception, the BAML team embraced an approach to solving complex operational problems that involves continuous evaluation and improvement across activities. It requires constant review and changes/improvements; for example, traditional onsite monitoring visits were modified to allow for ongoing remote monitoring and new reporting formats.

### Expertise and Quality

Master protocols, such as BAML, multiply the complexity of precision medicine protocols many fold. After initially reviewing 11 CROs that ranged in size/scale from large organizations to small boutique CROs, 6 CROs were invited for a proposal defense. The key criteria used for selecting a CRO to manage the study are listed in Table 1. Consequently, the expertise of the selected CRO in managing complex, blood cancer trials with novel and basic regulatory, quality assurance, project management, and monitoring functions have immensely benefited BAML.

**Table 1:** Key Selection Criteria for Contract Research Organization and e-Technology Product/Vendor

Category	Key criteria
Contract research organization (CRO)	Complex clinical trial management experience, preferably including Master Trials Deep oncology experience, specifically in AML Large and experienced staff with knowledge of cancer and low staff turnover (< 20%) Willing to partner for novel technology solutions outside of their core processes A shared commitment to the study design and process
Product/vendor	Product must have one or more of the features that BAML needs to manage the defined aspects of the Master Trial including complexity, size, data collection, transmission and accessibility, oversight, etc Product must have the ability to be integrated seamlessly and should be synergistic with other solutions that will be used for the trial Product must have configurability—ability to plug in a new or improved feature or make modifications without affecting already collected data or need revalidation Vendor must be willing to accept that BAML only use and pay for those features of the solution it needs for the trial Vendor must have an onboarding and training plan for internal (Sponsor) and external partners (CRO and sites) Vendor must be willing to provide support staff to assist in the product/software setup, implementation, and in resolving post-setup issues that come up during use, plus indicate any additional costs for these support services Vendor must be willing to customize product(s) for complex Master Trials and partner throughout the life of the program Vendor must be willing to offer non-profit costing

### New Technologies

The BAML team adopted and continues to add innovative e-technologies to help streamline clinical operations and facilitate oversight, data management and review, and thereby ensure data quality, safety and privacy. Similarly, several e-clinical solutions/vendors were initially evaluated. The key criteria used for product/vendor selection are listed in Table 1.

The selected e-technologies are summarized in Table 2 and discussed in the next section.

**Table 2:** Summary of e-Technology Platforms Deployed in the Beat AML Master Trial and Their Key Features

e-Technology platform	Key features
Clinical oversight platform	Central communication and collaborative tool Real-time and automated oversight: • Compliance summary • Engagement detail e-Learning management system for centralized training Streamlined safety report distribution and tracking Document distribution and tracking of readership: • Versioning • Commenting • Batch uploading Cloud-based 21 CFR Part 11 compliant ICH E6(R2) guidance supporting
e-Protocol/e-Source upload/EDC platform e-Protocol e-Source upload (e-SU) EDC	Advanced EDC/CDMS built specifically for oncology and complex study designs Digitizes the paper protocol within days Allows remote data monitoring Natively captures and exports data in SDTM format Data management Adept at protocol amendments and post production changes Combined with e-SU and DRP, allows safety signals to be reviewed and acted upon immediately Cloud-based 21 CFR Part 11 compliant
Data review platform (DRP)	Ingests data from different sources and EDC systems for clinical response and safety data reviews Seamlessly ingests and reports data in SDTM format
EHR-to-EDC application	Allows EHR patient data to be directly exported to the EDC of e-Protocol/e-Source upload/EDC platform and to other EDC systems Eliminates transcription errors and markedly reduces queries Cloud-based

*AML* Acute myeloid leukemia, *CFR* Code of Federal Regulations, *ICH* International Council for Harmonization of Technical Requirements for Pharmaceuticals for Human Use, *EDC* Electronic data capture, *CDMS* Clinical data management system, *SDTM* Standard data tabulation model, *EHR* Electronic health records

## e-Clinical Solutions Deployed in the Beat AML Master Trial

### Clinical Oversight Platform

The Clinical Oversight Platform (COP) was selected to be a central collaboration tool and key single point of communication across all Master Trial parties (vendors, sites, laboratories or biopharmaceutical companies) and that links the entire e-clinical ecosystem. As the BAML study materials are constantly changing, the COP serves as a “single source of truth” that assures the most current documents/information/training are always available. The COP has a site user-focused design, and provides a documented, data-driven oversight to improve participation, engagement and collaboration in clinical trials. The COP also has a lightweight, yet powerful electronic-Learning Management System (e-LMS) that allows centralized protocol training of site staff and has saved countless weeks of training per protocol had BAML used a traditional LMS. Also, the platform provides a streamlined safety report distribution and tracking system. Recently, the manufacturer of the COP developed a ‘compliance summary’ feature that was seamlessly plugged into the ecosystem and tracks compliance/engagement with study documents users should be aware of with a score, further demonstrating oversight. The COP is cloud-based, ICH E6(R2) guidance [27] supporting, and is 21 CFR Part 11 compliant.

### e-Protocol/e-Source Upload/Electronic Data Capture Platform

To assure seamless integration, a 3-in-1 next-generation EDC/clinical data management system, the e-Protocol/e-Source Upload/Electronic Data Capture (e-Protocol/e-SU/EDC) platform, built specifically for oncology and complex study designs, was selected for use in the BAML Master Trial. The e-Protocol/e-SU/EDC platform is cloud-based, 21 CFR Part 11 compliant, and the EDC supports all 5 modalities of e-source data acquisition as described in the FDA e-source guidance [28].

*e-Protocol for Protocol Dissemination and Management:* With BAML’s 13 sub-studies, each with 2 to 4 amendments per year, the logistical task of disseminating and managing the protocols was challenging, especially given the complexity of tracking protocols approved for each site and ensuring that the appropriate version was used at all times by each site. To ease these complexities, BAML decided to use a digital solution, an electronic protocol (e-protocol), developed by the same manufacturer of the e-Protocol/e-SU/EDC Platform. The paper protocol (available in the COP) is converted into an e-protocol (digitized) within days, the first to digitize protocols within days, and provides a consistent representation of each IRB-approved protocol version: arm/cycle/visit flow, visits and procedures. The e-protocol tool is centrally managed by the CRO project manager who can assign the appropriate versions to sites based on their individual IRB approvals. The e-protocol can then be accessed from any device (phone, tablet, desktop) and is used throughout the consenting process.

*e-Source Upload for Remote Clinical Monitoring:* On-site monitoring is resource intensive and creates significant time lags. This is amplified in the context of a program like BAML that requires current data for safety review and decisions across multiple protocols and multiple sponsor safety/pharmacovigilance (PVG) teams. Additionally, risk-based monitoring approaches are not yet well suited to studies with relatively small patient numbers (per study) in complex oncology settings. BAML wanted the ability to perform remote monitoring and data reviews in near real-time against both native source data (i.e., medical chart) and tabular data (i.e., electronic case report form [e-CRF]). In BAML, we selected the e-SU (in e-Protocol/e-SU/EDC platform) and a Data Review Platform as the technology solutions that best meet those operational requirements. The e-SU allows for the upload of unredacted electronic source documents, via certified copies of each data point to enable remote data monitoring. Redacting source data (i.e., removing all patient identifiers) is time-consuming and error prone. The e-SU solution partitions access to source data and allows monitors to review the unredacted data in read-only mode. The process fits into data transcription processes used by the site and is described in the patient informed consent. The e-SU is unique because just by navigating through the solution, metadata is “auto-tagged” to each uploaded document and logged in the audit trail. Since this process is automated, data does not need to be reconciled or re-monitored at a later date, as is the case with other solutions. For tabular review of the data, BAML uses standard data output and listings from the EDC of the platform.

*Electronic Data Capture for Data Management:* The advanced EDC of the e-Protocol/e-SU/EDC platform is used for electronic data capture/data management. Traditional EDC systems were not designed for complex oncology trials, including MPs, where studies have multiple arms and are constantly undergoing amendments, and they do not capture data in Standard Data Tabulation Model (SDTM) format. Also, migrations and ongoing data cuts for interim analyses are the norm in Master Trials. Therefore, newer and advanced EDC systems, such as the e-Protocol/e-SU/EDC platform, that are adept at amendments, allowing for rapid CRF changes (in hours) and which provides data in SDTM are required. This avoids the costly data transformations that take weeks to produce with the traditional EDC systems. The EDC in the e-Protocol/e-SU/EDC platform exports operational, clinical and safety data in real-time in submission-ready SDTM as well as via the application programming interface (API) backend. Data are therefore available for statistical analysis and dashboard reporting seamlessly and in real-time.

### Data Review Platform

To help with broader and systematic efficacy and safety review of the data, BAML also uses the DRP, which has the ability to ingest data from multiple EDC systems, including the core EDC of the e-Protocol/e-SU/EDC platform for review. The DRP also allows data from the different sub-studies and sources to be brought into a single interface with consistent data formats, allowing for the review of data, abnormal laboratory values, generation of reports and assessing sites’ performance relative to others. Based on nightly refresh of the data, the DRP generates interactive listings and reports for the Medical Advisory Committee to be used for the review of clinical response (efficacy) and safety data in near real time.

To further accelerate the generation of analysis data sets and regulatory submissions, BAML mandated the use of SDTM format, an international standard for clinical research data which is approved by the Food and Drug Administration (FDA) as a standard electronic submission format. Beat AML has now adopted the e-Protocol/e-SU/EDC platform as its core EDC software because it natively collects and exports data in SDTM, while the DRP also seamlessly ingests and reports data in SDTM-another example of course correction/improvement in program execution.

In summary, the combination of the e-Protocol/e-SU/EDC platform, DRP and technologically savvy data operations experts at the CRO, allows BAML to have centralized sponsor oversight and control over the entire data collection process. These innovative technology solutions help the sites perform their activities closer to actual visit dates, allows for near real-time clinical and safety data oversight, and facilitates interim data analyses and regulatory submissions. BAML leveraged newer “e-clinical” technologies, the COP and e-Protocol/e-SU/EDC platform, as well as the more established DRP to accelerate the first 2 years of the program by providing: easy-to-use communication tools, centralized training and organized training documentation, e-Protocols for easy versioning and staggered site rollout, and EDC with certified e-Source copies.

### Electronic Health Record to EDC Data Collection

In BAML’s third year, we onboarded a game-changing technology, also cloud-based and developed by the manufacturer of the e-Protocol/e-SU/EDC platform, the electronic health record-to-electronic data capture (EHR-to-EDC) application. The solution allows the transfer of up to 70 percent of a patient’s data directly from a site’s EHR system into the EDC system; exporting the data at the click of a button into the EDC (used for the first BAML sub-studies) or the EDC of the e-Protocol/e-SU/EDC platform (used for the later sub-studies). This markedly reduces data entry performed by staff onsite, the number of queries they receive as there are no transcription errors on data transferred via the validated application. The technology also leverages Natural Language Processing and other AI tools to further reduce the data entry burden placed on research sites, further reducing errors.

## Safety Review of Study Data

Given BAML’s complexity, we need real-time safety information across the entire study (Master and 13 sub-study protocols) as ongoing safety data review is at the core of the oversight of any clinical trial. Before onboarding a biopharmaceutical partner into the BAML program, LLS signs a harmonized PVG agreement with the partner that establishes reporting requirements. The Chief Medical Officer for BAML holds weekly safety calls with investigators and coordinators from the participating sites. Every actively enrolled subject is reviewed during this call and the information is captured in the weekly safety updates that are distributed to the investigators. We continuously improve the management and reporting of the weekly safety updates.

## Potential Advantages of the Operational Approaches and New Technologies

The use of these e-technologies allows ongoing oversight, and visualization and sharing of study data by all partners/stakeholders facilitating the development of operational metrics. The e-LMS of the COP allows centralized protocol training saving weeks of training per protocol had a traditional LMS been used. The engagement detail and compliance summary features of the COP ensure compliance in engaging with study information, and the batch uploading feature allows document sharing in seconds instead of countless streams of emails, resulting in time-saving and/or intangible benefits. The e-Protocol digitizes paper protocols within days and not months. The auto-tagging feature of e-SU allows remote data monitoring thereby eliminating delays in clinical research associate (CRA) monitoring and potentially the costs to both sites and CRO. The EDC is adept at amendments allowing for rapid CRF changes in hours. Furthermore, the EDC natively collects and exports data in SDTM facilitating interim data analysis, statistical data analysis and regulatory submissions. The marked reduction in data entry by site staff due to the EHR-to-EDC application will result in no transcription errors in the EHR data transferred and hence, in reduced query resolution time. Operational, clinical and safety data are exported in real-time by the EDC. This, combined with the e-SU and DRP allows safety signals to be viewed and acted upon immediately. Contracting with an experienced CRO partner and functional service providers (vendors), affords BAML the ability to access/use all these e-technologies without having to establish an in-house information technology infrastructure. By how much the above tangible and intangible benefits, potential advantages and the operational efficiencies gained will result in reduction in development time and/or in overall study costs is not currently known as the BAML trial is still ongoing.

## Challenges and Lessons Learned

Introducing new operational approaches and e-technologies come with their challenges. With the COP, the main challenge has been to remind study users to upload information onto the platform, to review it there and to use the solution as the main communication dashboard for the program. The main challenge for the sites with e-SU was adapting their data entry workflow and uploading source data to the system. Additionally, the CRO’s CRAs and data management personnel had to adapt their processes to leverage e-SU, which they did earnestly. The DRP challenges proved relatively minor and were limited to operational teams having to integrate data with consistent formats from different sources.

The lessons learned from the other challenges we faced and the approaches adopted to deal with them are:

- sites may resist the new e-technologies initially because of the perceived potential to increase workload, decrease productivity and time required for training. This was overcome by using “technology change management” strategies: working directly with sites to train the staff on how to use the “systems” and stressing the benefits of the e-technologies. Sites may require additional grants to on-board;
- new e-technologies even with onboarding and training had their toll initially. Some sites had a steeper learning curve which was reduced by effective training using super users at each site. Consequently, allowance should be made for potential delays in study timelines;
- for a central communication/collaborative platform to be successfully implemented in a complex trial like the BAML Master Trial, all stakeholders—site, CRO and Sponsor staff must upload data and information frequently onto the platform;
- a documented implementation plan, guidelines and approach to rollout of the new e-technologies for sites, CRO and Sponsor staff, with input from the vendor who may have previous experience doing this will help facilitate the adoption of the new e-technologies;
- ensure all key players are involved in the onboarding and training efforts including, physicians, coordinators, project managers, CRAs and backup staff at the sites and CRO, in case a staff calls out sick; and
- to continuously operationally improve a Master Trial of BAML’s magnitude, significant time should be spent re-evaluating, improving and re-inventing operational approaches.

## Role of the Regulator

While developing the BAML trial, the FDA has provided us with close ongoing regulatory guidance. Independent of our program, in July 2018, the FDA issued a guidance on the use of EHR data (regarding use of electronic systems and cloud-based technologies) in clinical investigations [29], followed by a guidance on MPs [5]. These guidance documents helped clarify our approaches to validating our solutions and source data verification requirements.

Earlier, in an effort to streamline and modernize clinical investigations, FDA developed a guidance entitled “Electronic Source Data in Clinical Investigations” [28], to be used together with the FDA guidance on computerized systems used in clinical investigations [30], to encourage and facilitate capturing source data in electronic form to ensure reliability, quality, integrity and traceability of data from electronic source to electronic regulatory submission.

## Next Steps

In line with BAML’s 5 core operational strategies and to further improve the implementation of the Master Trial, we are tackling a few new initiatives. First, while the central scientific committee constantly monitors for safety signals and patterns in the data collected for each sub-study, there has been limited data review across sub-studies. In order to reliably pool and visualize data across studies, standard endpoints, data definitions and structures are needed. Through discipline and expertise from the CRO’s data management team (using common SDTM mappings), we are now able to pool data across studies and look for patterns that might transcend the specific genetic profiles of our sub-populations.

As we continuously monitor for safety and clinical signals across sub-studies, it has become apparent that having operational insights across sub-studies is equally important. More recently, BAML has begun partnering with a data analysis technology company, to develop new Key Risk Indicators (KRIs) for genomically based, complex Master Trials within each sub-study and across sub-studies. With this approach, we are leveraging APIs and other automated integration points to seamlessly pull the data from the various sub-studies and technology solutions (EDC, clinical trial management system, labs, etc.), process them via a centralized risk algorithm, and present KRIs and other data visualizations for program management. The first version of the KRI dashboard was successfully delivered in July 2019.

## Conclusions

In conclusion, the operational strategies and e-technologies, and the expertise of the CRO have facilitated the conduct and better management of the Master Trial. The COP, a central communication/collaborative tool allows the complexity and size of the trial to be better managed while optimizing oversight; e-Protocol and e-LMS allow paper protocols to be digitized within days and centralized protocol training, respectively, saving weeks of training per protocol; e-SU uploads certified copies of unredacted source documents allowing remote data monitoring; the EDC captures and exports data in real-time in analysis- and submission-ready SDTM, and allows rapid e-CRF changes in hours post-amendments; EHR-to-EDC markedly reduces data entry at the sites and with no transcription errors, reduces the number of queries received at the site; and the DRP integrates data from different sources with consistent formats for review and generation of reports. Overall, the combined activities of the different components of the COP, e-Protocol/e-SU/EDC platform and the EHR-to-EDC allows Sponsor oversight and control over the entire data collection process, streamline and improve data collection at the sites and enhance data transmission, accessibility and review, while maintaining data privacy and compliance. We believe the challenges and lessons learned from the approaches developed in this complex Master Trial can potentially accelerate drug development and expedite the achievement of our goal of improving clinical outcomes, and can provide insights to those conducting Master Trials and adopting e-technologies.
